# Noninvasive Neurally Adjusted Ventilator Assist Ventilation in the Postoperative Period Produces Better Patient-Ventilator Synchrony but Not Comfort

**DOI:** 10.1155/2020/4705042

**Published:** 2020-06-20

**Authors:** L. O. Harnisch, U. Olgemoeller, J. Mann, M. Quintel, O. Moerer

**Affiliations:** ^1^Department of Anaesthesiology, University Medical Centre Göttingen, Germany Robert-Koch-Str. 40 D-37099 Göttingen; ^2^Department of Cardiology and Pulmonology, University Medical Centre Göttingen, Robert-Koch-Str. 40 D-37099 Göttingen, Germany

## Abstract

**Background:**

Noninvasive neurally adjusted ventilatory assist (NAVA) has been shown to improve patient-ventilator interaction in many settings. There is still scarce data with regard to postoperative patients indicated for noninvasive ventilation (NIV) which this study elates. The purpose of this trial was to evaluate postoperative patients for synchrony and comfort in noninvasive pressure support ventilation (NIV-PSV) vs. NIV-NAVA.

**Methods:**

Twenty-two subjects received either NIV-NAVA or NIV-PSV in an object-blind, prospective, randomized, crossover fashion (observational trial). We evaluated blood gases and ventilator tracings throughout as well as comfort of ventilation at the end of each ventilation phase.

**Results:**

There was an effective reduction in ventilator delays (*p* < 0.001) and negative pressure duration in NIV-NAVA as compared to NIV-PSV (*p* < 0.001). Although we used optimized settings in NIV-PSV, explaining the overall low incidence of asynchrony, NIV-NAVA led to reductions in the NeuroSync-index (*p* < 0.001) and all types of asynchrony except for double triggering that was significantly more frequent in NIV-NAVA vs. NIV-PSV (*p* = 0.02); ineffective efforts were reduced to zero by use of NIV-NAVA. In our population of previously lung-healthy subjects, we did not find differences in blood gases and patient comfort between the two modes.

**Conclusion:**

In the postoperative setting, NIV-NAVA is well suitable for use and effective in reducing asynchronies as well as a surrogate for work of breathing. Although increased synchrony was not transferred into an increased comfort, there was an advantage with regard to patient-ventilator interaction. The trial was registered at the German clinical Trials Register (DRKS no.: DRKS00005408).

## 1. Introduction

Noninvasive ventilation (NIV) is increasingly used for COPD (chronic obstructive pulmonary disease) and acute respiratory insufficiency [1], in home mechanical ventilation [2], and postextubation care [3]. However, several mechanisms like poor tolerance, mask-related complications, severity of the underlying disease, but also poor patient-ventilator interaction can lead to NIV failure [4, 5].

Asynchrony between patient and ventilator is a common finding and underestimated problem [6] being an important cause for NIV failure [7], since it might actually increase work of breathing [8]. Signs of impaired patient-ventilator interaction are ineffective triggering and double-triggering where the ventilator either does not support a breath at all or one inspiratory effort is sensed as two and supported by the ventilator as such, resulting in hyperinflation of the lung, respectively.

During neurally adjusted ventilatory assist (NAVA), an adaptive ventilator mode, the ventilator respiratory cycle and a proportional ventilatory assist are based on the electrical activity of the diaphragm (EAdi) detected via an oesophageal catheter ([9]). Thus, during NAVA airway pressure, ventilator system flow, intrinsic PEEP (positive endexpiratory pressure), lung volume, and airleaks only marginally influence ventilator cycling [10]. NAVA is able to significantly reduce respiratory workload and asynchrony during invasive as well as noninvasive ventilation [11–13]. Furthermore, by way of proportionally assisting a patient's breath rather than assisting it in a not-changing manner for each breath, NAVA also exercises lung-protective ventilation. A recent meta-analysis revealed that NIV-NAVA significantly enhances patient-ventilator interaction and lowers severe asynchrony compared to NIV-PSV[14]. However, the effects on synchrony and patient comfort in previously lung-healthy patients needing NIV for postextubation care have not been evaluated. NIV-NAVA in these subjects is of special interest in some regards: first, reduced asynchrony may accelerate the improvement of gas exchange and reduce ICU- and hospital length-of-stay. Second, NIV-NAVA is an elegant way to ensure lung-protective ventilation [15], which may often not be thought of in these subjects or is admittedly often difficult to follow due to the dynamic situation of postoperative respiratory failure. Therefore, we decided to target postoperative subjects, because lung-protective ventilation is critical in these patients as well and increased synchrony with accelerated improvement in gas exchange may reduce the length of ICU- and hospital stay.

The objective of this trial was to evaluate if NIV-NAVA would improve patient-ventilator synchrony measured by NeuroSync-index [16] (primary outcome) as well as perceived patient comfort (secondary outcome) compared to NIV-PSV. We conducted a prospective randomized crossover observational trial and assessed differences in patient comfort and patient-ventilator interaction between noninvasive pressure support ventilation (NIV-PSV) and NIV-NAVA in previously lung-healthy adult subjects in the postoperative period.

## 2. Material and Methodes

The study was conducted in an adult surgical ICU (intensive care unit) of a tertiary medical centre in 2012/13 and 2016 and designed as a single-blind prospective randomized crossover observational trial. This study was approved by the Research Ethics Board of Georg-August University Goettingen (#19/1/12), and written informed consent was obtained from all subjects participating in the trial prior to data acquisition. The trial was registered at the German Trials Register (http://www.drks.de; DRKS00005408, date of registration: 30.10.2013).

Subjects were eligible if attending physicians indicated postextubation noninvasive ventilation. After informed consent, the EAdi-catheter was placed as described elsewhere [17], and the facemask was tightly strapped to the subject's face (AcuCare™ F1-0 Hospital NV Full Face Mask, ResMed, Martinsried, Germany). Subjects received both ventilation modes successively but were randomized to the initial mode and were kept unaware of the actual mode. We used the Servo-I ventilator (Maquet, Getinge Group; Rastatt, Germany) as the only ventilation device throughout the whole trial. Before ventilation was started, RASS Score (Richmond Agitation and Sedation Scale) was documented and correct catheter position was verified using the EAdi positioning tool [17]. Ventilator settings were at the discretion of the attending physician with respect to the subject's clinical status; PEEP was set according to the low PEEP/FiO_2_ table as proposed by the ARDS Network.

Measurements were started after stable conditions were achieved. We recorded a total of 20 minutes for each ventilation mode, every five minutes clinical parameters (heart rate, blood pressure, respiratory rate) as well as ventilation parameters (fraction of inspired oxygen, tidal volume, PEEP, maximum inspiratory pressure, NAVA-level/pressure support, EAdi-peak) were documented (ESM figure 1). Ventilator parameters as well as flow and pressure curves were extracted through an interface at a sampling rate of 100 Hz using a dedicated software (Servo-Tracker V4.1, Maquet, Getinge Group, Solna, Sweden). Acquired data were transferred to a dedicated software (NeuroVent Analysis Software, Research Inc., Toronto, ON, Canada) measuring in- (TI) and expiratory time (TE), trigger and off-cycling delays, and negative pressure duration (defined as the time from the pressure drop below PEEP level up to the moment when PEEP level is reached again by pressure support) by placing cursors in the ventilator tracings and calculating differences between them during six continuous minutes of the recording (ESM table 1, ESM figure 2 and “cursor placement”). Ventilator tracings were also assessed for ineffective efforts, double-triggering, and autotriggering [16].

Based on the assessed variables, asynchrony-index [16] (asynchrony − index [%] = number of asynchrony events/total respiratory rate [ventilator cycles + wasted efforts] × 100) and NeuroSync-index ([18]) (average of all absolute values for the errors [both-sided trigger-on and cycle-off errors] for all events) were calculated manually. Classification of asynchrony was used as defined before: asynchrony index >10%; NeuroSync-index >33% = dysynchrony, 100% = asynchrony.

After fifteen minutes of ventilation, an arterial blood gas was drawn and subjects were questioned about comfort and quality of ventilatory support. The questionnaire consisted of five closed-ended questions and two open-ended questions about the general experience of ventilation, where a visual analogue scale (VAS), was used for each ventilation mode received. The VAS was charted in centimetres starting from the left side (low values, very bad comfort) (ESM figures 3 and 4).

Questions to the subjects at the end of each session:

- Does the ventilation via facemask facilitate your breathing? Yes; no
- Do you feel your exhalation is impaired? Yes; no
- Does the ventilator react too fast/too slow? (Does the ventilator adapt to your breathing?) Yes; no
- Do you get too much/too little air? Yes; no
- Do you feel the mask does not seal properly/too much air leaks? Yes; no
- How comfortable do you feel? VAS-scale (one for each ventilation mode)

### 2.1. Statistical Analysis

Statistical analysis was performed using SPSS 25.0 (Statistical Package for Social Sciences, International Business Machines Corporated [IBM], Armonk, New York, USA). Data were tested for normal distribution using Shapiro-Wilk test. Parametric variables were analysed by paired *t*-test, nonparametric variables were analysed by Wilcoxon signed-rank test. Variables were further analysed by two-way-repeated-measure ANOVA (analysis of variance) with Bonferroni post hoc correction with respect to the subject “mode” to account for the cross-over design. Quantitative parameters were transformed onto a nominal scale (yes = 1, no = 0) and analysed using Fishers exact-test. Results are presented as mean and standard deviation (SD) for parametric and median and interquartile range (IQR) for nonparametric variables; level of significance was assumed at *p* < 0.05.

This manuscript adheres to the applicable CONSORT guidelines.

## 3. Results

All subjects had a RASS Score of zero and did not receive sedation/analgesia throughout the study period; subject characteristics and ventilator settings are shown in Tables 1 and 2, respectively. Blood gas parameters did not differ between modes (ESM table 2).

NIV-NAVA led to shorter inspiratory delay (NIV-PSV 186 ± 81 ms vs. NIV-NAVA 50 ± 36 ms; *p* < 0.001) as well as expiratory delay (NIV-PSV −48 ± 85 ms vs. NIV-NAVA 10 ± 14 ms; *p* = 0.003).

**Figure 1:**
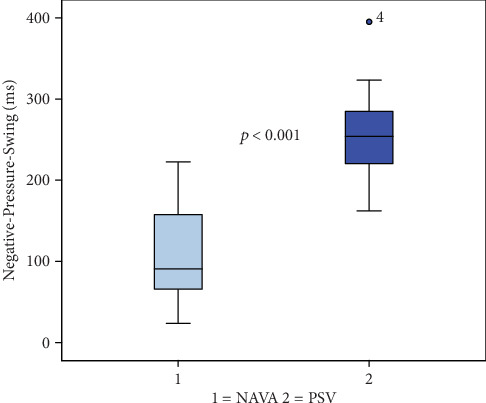
Whiskers-boxplots of negative pressure swing as a surrogate for work-of-breathing.

Inspiratory and expiratory time did not differ (TI: NIV-PSV 1171 ± 314 ms vs. NIV-NAVA 1138 ± 383 ms; *p* = 0.564; TE: NIV-PSV 2268 ± 800 ms vs. NIV-NAVA 2074 ± 770 ms, *p* = 0.130; TTOT NIV-PSV 3429 ± 1076 ms vs. NIV-NAVA 3222 ± 1094 ms; *p* = 0.353). Negative pressure duration showed statistical significance favouring NIV-NAVA (NIV-PSV 253 ± 55 ms vs. NIV-NAVA 108 ± 58 ms; *p* < 0.001) (Figure 1, Table 3).

**Figure 2:**
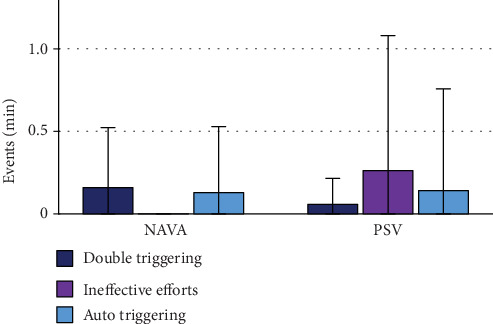
Frequency of asynchrony sorted by type; modes contrasted. Differences showed statistical significance for double triggering (*p* = 0.02) and ineffective efforts (*p* = 0.001), but not for auto triggering (*p* = 0.584).

Double triggering was more frequent in NIV-NAVA (NIV-PSV 0.06 ± 0.08/min vs. NIV-NAVA 0.16 ± 0.18/min; *p* = 0.02). While auto triggering was the same in both modes (NIV-PSV 0.15 ± 0.43/min vs. NIV-NAVA 0.13 ± 0.24/min; *p* = 0.584), ineffective efforts did not appear in NIV-NAVA but in NIV-PSV (NIV-PSV 0.26 ± 0.62/min vs. NIV-NAVA 0 ± 0/min; *p* = 0.001) (Figure 2). Asynchrony-index was not different in NIV-NAVA vs. NIV-PSV (NIV-PSV 2.43 ± 3.12% vs. NIV-NAVA 1.58 ± 1.89%; *p* = 0.258), whereas NeuroSync-index was significantly less in NIV-NAVA compared to NIV-PSV (NIV-PSV 18.22 ± 8.19% vs. NIV-NAVA 9.21 ± 5.85%; *p* = 0.001).

Two-way-repeated-measure ANOVA revealed no significant interaction regarding the order in which the modes were used (*p* = 0.06) (ESM table 3).

Questionnaires were analysed regarding comfort of NIV-NAVA vs. NIV-PSV. Most subjects found NIV helpful, (question one) (NIV-NAVA 70%, NIV-PSV 68%, *p* = 0.293) and about half of the subjects denied impeded expiration (question two) (NIV-NAVA 53%, NIV-PSV 53%, *p* = 0.351). Asked for the subjective feeling of premature or delayed cycling most subjects answered “no” (NIV-NAVA 57%, NIV-PSV 73%, *p* = 0.192) and most subjects did not feel the flow to be insufficient (NIV-NAVA 61%, NIV-PSV 71%; *p* = 0.325). The general impression of the ventilation mode did not differ between modes (VAS: NIV-PSV 5.31 ± 2.36 cm, NIV-NAVA 5.41 ± 2.31 cm; *p* = 0.907).

## 4. Discussion

Our results concur with most recent studies in that NIV-NAVA improves patient-ventilator interaction compared to pressure support ventilation in all measures of asynchrony except for double triggering [12, 19–25].

During NIV, the ventilator is triggered by electromyographic signals, this is advantageous because it antagonizes the devastating effect of leakage. In an early study in healthy volunteers receiving NIV-NAVA via a helmet interface, the advantages of a neural trigger were revealed [26] . Our data, in concert with recent studies [22], confirm this effect showing an inspiratory delay decidedly shorter for NIV-NAVA than for NIV-PSV.

Expiratory delay was also reduced during NIV-NAVA compared to NIV-PSV as reported before ([11, 22, 24]); although, the delays we found were substantially shorter than previously reported. The most prevalent difference is the set off-cycling value during NIV-PSV (our trial 40 ± 7.32%, Schmidt et al. 30%, Ducharme-Crevier not given, probably default 30%, Longhini 35%), whereas during NIV-NAVA the ventilator cycles off at 70% EAdi-peak as fixed by the manufacturer. Our data support evidence that synchrony can be improved in NIV-PSV by meticulous adjustments of ventilator settings, although with a limited effect [27].

Double triggering was more prevalent in NIV-NAVA than in NIV-PSV [21, 25]. Other investigators report double-triggering to be caused by an EAdi wave most likely due to sighs or periodic neural respiratory hyperactivity [12, 21] which can be confirmed from our tracings. One might explain these sighs to be due to relative insufficient inspiratory flow. Since the inspiratory flow in NIV-NAVA is proportional to EAdi-slope, the subject might augment a relative too small inspiratory flow due to normal respiratory variability by a consecutive sigh. This phenomenon is less likely in NIV-PSV because the inspiratory flow is usually high, sometimes even too high [28]. Moreover, we also found a lesser rate of double triggering in NIV-PSV than shown before [16].

Ineffective efforts are usually very frequent in PSV which is most likely due to the fact that they are the common final path of a lot of suboptimal ventilator settings (inspiratory trigger too high or too low, pressure support too high or too low) [16, 19, 21, 29]. Fifty percent of our subjects experienced ineffective efforts in NIV-PSV, which is less than described by others [30] but still unacceptably high. With NIV-NAVA, ineffective efforts can be avoided completely as has consistently been shown [11, 19, 30, 31].

To globally evaluate for asynchronies during ventilator assisted breathing indices such as asynchrony-index and NeuroSync-index have been proposed ([16], [18]). Compared to others, we found lower indices [14, 24] most likely due to our study population which was previously lung healthy.

**Figure 3:**
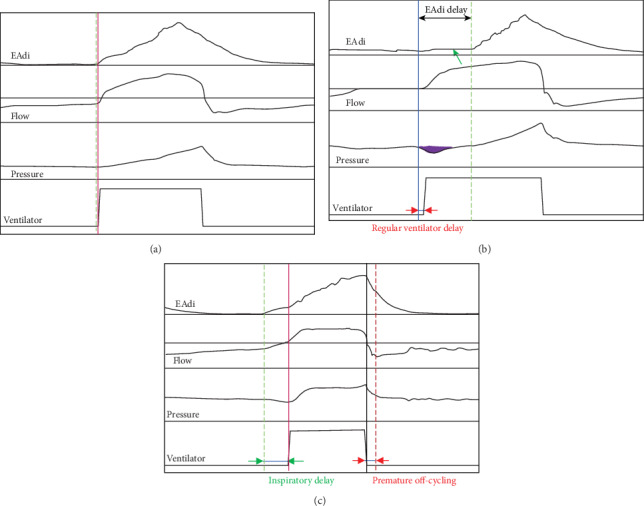
Presented are three different screenshots of ventilator tracings; tracing on top always EAdi, second tracing flow, third tracing pressure, fourth tracing ventilator signal. The axes are as follows: EAdi in microvolt, flow in liters per minute, pressure in mbar, ventilator dichotomous off/on, *y*-axes: time in milliseconds. Values are spared intentionally because this figure should be seen as a schematic rather than a visual representation of study data. (a) Perfect neuronal triggering, the ventilator cycles on (red vertical line) at exactly the same time as the increase in EAdi signal tracing (green broken vertical line) happens; times not marked because in this perfectly triggered breath there is no delay. (b) Visual presentation of the concept of “EAdi true”. The time point marked with the black double-arrow is the moment when a decrease in pressure happens but the EAdi tracing does not show an increase, rather does it show the plateau mentioned in the text, representing a blacked out ECG signal (green arrow). During the plateau inspiratory activity of the diaphragm cannot be detected. The start of inspiration is therefore not where the EAdi tracing starts to rise, but rather where a drop in pressure tracing during the EAdi-plateau is detected. This is where the EAdi-signal would rise, if ECG “disturbance” had not happened. EAdi-delay: black double-arrow; regular ventilator delay: red arrows; purple area: negative pressure swing. (c) Pneumatically triggered breath during PSV, visual presentation of inspiratory and expiratory delay. The beginning of the EAdi tracing representing the start of inspiration is marked by the vertical broken green line. The start of ventilator activity (red vertical line) happens belated relative to EAdi but perfectly in time in regard to PSV criteria. Without the EAdi tracing this inspiratory delay cannot be detected. The end of ventilator activity (black vertical line) happens before EAdi tracing has reached the off-cycling criterion (vertical broken red line, 70% EAdi-peak); at this moment, the patient's diaphragm was still activated denoting that inspiration was still ongoing. Expiratory delay is marked by red arrowhead.

A successful (i.e., synchronous) patient-ventilator interaction leads to on-time triggering and adequate ventilatory support and is therefore effective in unloading respiratory muscles. Respiratory muscle workload is often measured as (inspiratory) pressure-time-product (PTP), by oesophageal pressure or as P0.1 (airway occlusion pressure 0.1 seconds after the start of inspiratory flow). All of these methods have their drawbacks [32–34]. Due to the clinical situation, being limited to assess work of breathing invasively during NIV, we evaluated respiratory muscle workload by calculating negative pressure duration which corresponds approximately to the work performed by the respiratory muscles to reach the trigger threshold (purple area in Figure 3). Ideally, during NAVA, this value would be zero, because the ventilator should start support exactly when the diaphragm contracts. We found a negative pressure duration that was significantly shorter for NIV-NAVA than for NIV-PSV which resembles results those of other trials concluding that NIV-NAVA is more effective with regard to unloading respiratory muscles than NIV-PSV [31].

NAVA mode has a pneumatic trigger safety back-up intended for situations of catheter-failure or displacement following the “first-come-first-serve” principle. In other trials, one-fifth to one-third of breaths in NAVA was triggered pneumatically [13, 31] (our trial: 29.7%). Possible explanations are displaced catheters or a modified activation of respiratory muscles, yet another explanation seldomly addressed is the ECG-filter of the NAVA mode [18]. By means of proximity of the catheter to the heart, ECG signals are part of the electrical recording and are actually used in the catheter positioning tool [17]. Since ECG amplitude might be high in relation to the electromyographic signals, a complex filtering algorithm has been developed [35]. While filtering during the rise in the EAdi signal can be compensated, a filtered signal during the triggering phase might cause (negative) trigger delays specifically related to the NAVA mode [18] (Figure 3).

To account for this systematic failure, we placed the “EAdi-true” cursor; we are aware that the name of this cursor suggests an absoluteness that it might not have. However, the assumption of ECG interference might be false if accessory ventilatory muscles generate an inspiratory pressure earlier than the diaphragm, for example, in case of ICU acquired weakness syndrome. In our study, the value for the inspiratory delay without EAdi-true would be 50 ms which is more than half that of what we tend to call the true delay (111 ms). Clinical usefulness of this corrected inspiratory trigger can be questioned because no measure can be taken to account for it. However, studies on NAVA with regard to patient-ventilator interaction should report this value, as well as the amount of pneumatically triggered breaths.

Our study has some limitations: we studied a small group of previously healthy subjects being clinically indicated by attending physicians to receive postextubation NIV. Probably most of these subjects would have done well without NIV, and therefore, they might not have experienced the need for ventilatory support. None of our subjects had experience with NIV, so other than subjects receiving NIV regularly like patients suffering from COPD our subjects might not have felt subtle differences a more experienced NIV-receiver might have recognized. Furthermore, we did not measure leakage during the study period. We only clinically minimized leak by tight mask fitting and checked and adjusted mask fitting with regard to leak due to our standard-of-care which includes keeping leakage below 30%.

We hypothesized that patient comfort would be higher during NIV-NAVA. This effect has been shown in children [12] and in adults at risk for extubation failure ventilated via helmets [20], as well as in healthy subject by [36]. In agreement with two other studies [19, 22], we were not able to show a difference in comfort between NIV-PSV and NIV-NAVA. One possible explanation is the difference in need for NIV, where a subject with a high demand for ventilatory assist will probably directly estimate the benefit of improved synchronization, whereas a subject that might also benefit from simple NIV-CPAP (continuous positive airway pressure) might not. Especially in case of subjects not used to NIV, felt differences between the modes are minute and might easily be overshadowed by environmental factors. Another possible explanation is the short time of application to compare the two modes; with longer application time, a positive effect of improved synchrony on comfort might have become evident.

A strength of our study is that we used the same ventilator for both modes; nevertheless, our results may not be automatically applied to other ventilators.

## 5. Conclusion

NAVA used in noninvasive ventilation is able to safely increase its efficiency in postoperative, previously lung-healthy subjects. Our postoperative subjects did not experience improved comfort with NIV-NAVA vs. NIV-PSV. However, the reduction of asynchrony is an argument to use NAVA in noninvasive ventilation; although, a larger randomized controlled trial that targets intubation rates and outcome with NIV-NAVA compared to conventional NIV-PSV is needed.

## Figures and Tables

**Table 1:** Individual patient characteristics including age, diagnosis ICU admission, type of surgery, and SAPS II on admission.

Included patient	Sex	Age (years)	Diagnosis (admission)	Type of surgery	SAPS II
1	Male	75	Endocarditis	SVR	66
2	Female	69	Aortic ulceration	Aortic arch replacement	41
3	Male	83	Combined aortic vitium, endocarditis	SVR	67
4	Male	60	Decompensated congestive heart failure	CABG	47
5	Female	71	Aortic and mitral valve stenosis	SVR	64
6	Male	74	Postoperative pneumonia	CABG	40
7	Male	76	Hemorrhagic shock	CABG	28
8	Male	74	Coronary artery disease	CABG	60
9	Male	41	Hemorrhagic shock	CABG	45
10	Male	67	NSTEMI	CABG	37
11	Male	68	Tricuspid valve insufficiency	SVR	44
12	Male	45	Multiple extremity fractures	Surgical bone repair	34
13	Male	51	Aortic dissection	Aortic arch replacement	66
14	Male	44	Aortic valve insufficiency	SVR	36
15	Female	57	Postoperative respiratory failure	CABG	42
16	Female	74	Coronary artery disease	CABG	39
17	Female	75	NSTEMI	CABG	35
18	Male	46	Aortic valve stenosis	SVR	29
19	Male	65	Coronary artery disease	CABG	39
20	Male	74	Coronary artery disease	CABG	46
21	Male	84	Lung mass	Diagnostic VATS	47
22	Female	73	Coronary artery disease	CABG	34

**Table 2:** Respiratory characteristics displayed as mean and standard deviation.

Parameter	Mean (SD)
Age (years)	65.7 ± 12.25
SAPS II on admission	44.82 ± 11.90
Ratio female: male	1 : 2.7
Catheter depth (cm)	64.29 ± 6.15
Pre-NIV pH	7.44 ± 0.04
Pre-NIV paO_2_ (mmHg)	88.70 ± 24.89
Pre-NIV paCO_2_ (mmHg)	41.55 ± 4.80
PEEP (cmH_2_O)	6.23 ± 1.07
F_i_O_2_	40.54 ± 4.54
Horowitz-index	170.99 ± 95.89
NAVA level (cmH_2_O/*μ*V)	0.77 ± 0.45
P_insp_ (cmH_2_O)	13.64 ± 2.46
Pressure support (cmH_2_O)	6.25 ± 2.29
Expiratory trigger NIV-PSV (%)	40 ± 7.32
Tidal volume (ml/kg IBW)	8.30 ± 1.86
Respiratory rate (bpm)	20.75 ± 6.74

**Table 3:** Presented are the mean respiratory times for NIV-NAVA and NIV-PSV, (SD), median (IQR), respectively; ∗significant difference.

	NIV-PSV	NIV-NAVA	
Inspiratory time (ms)	1171 ± 314	1138 ± 383	*p* = 0.564
Expiratory time (ms)	2267 ± 800	2074 ± 770	*p* = 0.130
Total respiratory time (TTOT) (ms)	3180 (3071-4047)	3289 (2832-3843)	*p* = 0.353
Negative pressure duration (ms)∗	253 ± 55	108 ± 58	*p* < 0.001∗

## Data Availability

The data can be supplied from the corresponding author on demand.

## Additional Points

Take-home message: NAVA used during NIV for postoperative respiratory support in lung-healthy patients effectively reduces asynchrony. NIV-NAVA actually reduces ineffective respiratory efforts to zero compared to NIV-PSV. Nevertheless, this does not translate into increased comfort.

## Supplementary Materials

Supplementary Materials ESM table 1: used delays and their calculation formula from the placed cursors. ESM table 2: results of ABG analyses (mean and SD) after fifteen minutes of ventilation. Differences are marginal and statistically not significant. ESM table 3: results of repeated measures ANOVA for main effect of “mode” and the interaction between mode and order, significance is marked∗. ESM figure 1: study design. ESM figure 2: tracings and cursors used in the study. For cursor description please refer to “cursor placement”, for definition of times and delays please refer to ESM table 1. ESM figure 3: questionnaire to assess patient comfort for each ventilation mode; original sheet for the first mode; for the second mode, the sheet is headlined, mode 2 “but otherwise just the same”. ESM figure 4: visual analog scale to evaluate overall impression of the ventilation mode, each mode was evaluated separately. The patient was given a pen and asked to draw a vertical line at the place where they feel their comfort level is. Translation: headline “noninvasive ventilation mode 1/ 2”; left means “very uncomfortable, wearing”, right means a very pleasant experience. These explanations were also given to the patient during the trial.
