# Supplementary material for: Noninvasive Neurally Adjusted Ventilator Assist Ventilation in the Postoperative Period Produces Better Patient-Ventilator Synchrony but Not Comfort
Source: Pulm Med. 2020 Jun 20;2020:4705042. doi: 10.1155/2020/4705042 (PMC7327603; doi:10.1155/2020/4705042)
Supplement: Supplementary Materials — ESM table 1: used delays and their calculation formula from the placed cursors. ESM table 2: results of ABG analyses (mean and SD) after fifteen minutes of ventilation. Differences are marginal and statistically not significant. ESM table 3: results of repeated measures ANOVA for main effect of “mode” and the interaction between mode and order, significance is marked∗. ESM figure 1: study design. ESM figure 2: tracings and cursors used in the study. For cursor description please refer to “cursor placement”, for definition of times and delays please refer to ESM table 1. ESM figure 3: questionnaire to assess patient comfort for each ventilation mode; original sheet for the first mode; for the second mode, the sheet is headlined, mode 2 “but otherwise just the same”. ESM figure 4: visual analog scale to evaluate overall impression of the ventilation mode, each mode was evaluated separately. The patient was given a pen and asked to draw a vertical line at the place where they feel their comfort level is. Translation: headline “noninvasive ventilation mode 1/ 2”; left means “very uncomfortable, wearing”, right means a very pleasant experience. These explanations were also given to the patient during the trial. [file 4705042.f1.docx]

| Delay | Cursor formula |
| --- | --- |
| TI (EAdi) | EndIEAdi - BegIEAdi |
| TE (EAdi) | EndEEAdi - EndIEAdi |
| TTOT (EAdi) | EndEEAdi - BegIEAdi |
| Td_insp_1 | Press0Pos - BegIEAdi |
| Td_insp_2 | Press0Pos - EAdiTrue |
| Negative Pressure Swing | Press0Pos - Press0Neg |
| Td_exsp_ | EndIPress - EndIEAdi |
| DelayResp1 | BegIVent - BegIEAdi |
| DelayResp2 | BegIVent - EadiTrue |
| DelayResp3 | EndIVent - EndIEAdi |

**ESM Table 1:** Used delays and their calculation formula from the placed cursors.

|  | **NIV-PSV** | **NIV-NAVA** |  |
| --- | --- | --- | --- |
| **pH** | 7.44 ± 0,046 | 7.44 ± 0,047 | p = 0.143 |
| **PaCO_2_ (mmHg)** | 41.77 ± 5,36 | 41.30 ± 5,75 | p = 0.105 |
| **PaO_2_ (mmHg)** | 93.50 ± 29.60 | 96.52 ± 34.42 | p = 0.275 |

**ESM Table 2:** Results of ABG analyses (mean and SD) after fifteen minutes of ventilation. Differences are marginal and statistically not significant.

|  | **Main effect „mode“** | **Interaction mode vs. order** |
| --- | --- | --- |
| **pH** | F (1.21) = 2.321, p = 0.143, η^2^ = 0.100 | F (1.21) = 0,389, p = 0.540, η^2^= 0.018 |
| **P_a_CO_2_** | F (1.21) = 2.865, p = 0.105, η^2^ = 0.120 | F (1.21) = 0.355, p = 0.558, η^2^ = 0.017 |
| **P_a_O_2_** | F (1.21) = 1.258, p = 0.275, η^2^= 0.057 | F (1.21) = 0.248, p = 0.623, η^2^ = 0.012 |
| **TI** | F (1.21) = 0.313, p = 0.582, η^2^ = 0.015 | F (1.21) = 3.433, p = 0.078, η^2^ = 0.140 |
| **TE** | F (1.21) = 2.575, p = 0.124, η^2^ = 0.109 | F (1.21) = 1.063, p = 0.314, η^2^ = 0.048 |
| **TTOT** | F (1.21) = 1.406, p = 0.249, η^2^ = 0.063 | F (1.21) = 2.408, p = 0.136, η^2^ = 0.103 |
| **NPS** | F (1.21) = 89.041, p < 0.001, η^2^ = 0.809***** | F (1.21) = 0.729, p = 0.403, η^2^ = 0.034 |
| **Td_insp_1** | F (1.21) = 48.710, p < 0.001, η^2^ = 0.699***** | F (1.21) = 1.916, p = 0.181, η^2^ = 0.084 |
| **Td_insp_2** | F (1.21) = 85.456, p < 0.001, η^2^ = 0.803***** | F (1.21) = 0.986, p = 0.332, η^2^ = 0.045 |
| **Td_exsp_** | F (1.21) = 11.244, p = 0.003, η^2^ = 0.349***** | F (1.21) = 0.612, p = 0.443, η^2^ = 0.028 |
| **DelayResp1** | F (1.21) = 15.808, p = 0.001, η^2^ = 0.429***** | F (1.21) = 0.906, p = 0.352, η^2^ = 0.041 |
| **DelayResp2** | F (1.21) = 46.999, p < 0.001, η^2^ = 0.691***** | F (1.21) = 0.005, p = 0.944, η^2^ = 0.000 |

**ESM table 3:** Results of repeated measures ANOVA for main effect of “mode” and the interaction between mode and order, significance is marked*.

***Cursor placement***

Cursors were positioned for every breath within the chosen timeframe on the following defined points in the EAdi, flow, pressure, and ventilator tracings; also see ESM figure 2:

EAdi tracing:

- “**BegIEAdi**”: beginning of inspiration; increase of curve, trigger threshold at 0.5 μV (dotted light green cursor)
- “**PeakIEAdi**”: vertex of curve (dotted light blue cursor)
- “**EndIEAdi**”: end of inspiration; decrease of curve to preassigned 70% of peak EAdi value (red dotted cursor)
- “**EndEEAdi**”: end of exspiration; new increase of EAdi curve (second dotted light green cursor)
- “**EAdi-True**”: neural beginning of inspiration in case of artificial overlap of the EAdi and the ECG tracing; the exact point of decrease of pressure tracing below PEEP-level (yellow cursor)

Flow tracing:

- “**BegIFlow**”: beginning of inspiration; increase of curve (broken light green cursor)
- “**EndIFlow**”: end of inspiration; decrease of curve (broken red cursor)
- “**EndEFlow**”: end of exspiration; new increase of curve (second broken light green cursor)

Pressure tracing:

- “**Press0Neg**”: beginning of patient’s inspiratory effort; decrease of pressure curve below PEEP-level (grey cursor)
- “**Press0Pos**”: beginning of assisted cycle; increase of curve back to PEEP-level (olive-green cursor)
  (NAVA: cursors “Press0Neg” and “Press0Pos” are ideally stacked)
- “**EndIPress**”: end of inspiration; decrease of curve (purple cursor)
- “**EndEPress**”: end of exspiration; new decrease below PEEP-level (bluish grey cursor)

Ventilator tracing:

- “**BegIVent**”: beginning of assisted cycle; increase of curve (pink cursor)
- “**EndIVent**”: end of assisted cycle; decrease of curve (black cursor)

The inspiratory periode was calculated from the difference of the beginning and the end of the inspiratory EAdi signal. The exspiratory period was calculated from the difference of the beginning and the end of the exspiratory EAdi signal.

Delayed inspiratory triggering was determined as the difference between the increase above PEEP-level (Press0Pos) and 1) the initial increase in EAdi signal and 2) the EAdi-true.

Exspiratory delay was estimated from the difference between the end of the inspiratory EAdi signal and the decrease of the pressure curve. Furthermore the negative pressure swing was calculated from the difference between the pressure drop blow PEEP-level and the new increase above this level.


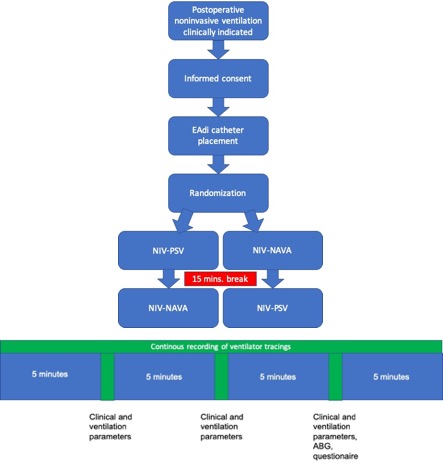


**ESM figure 1**: Study design


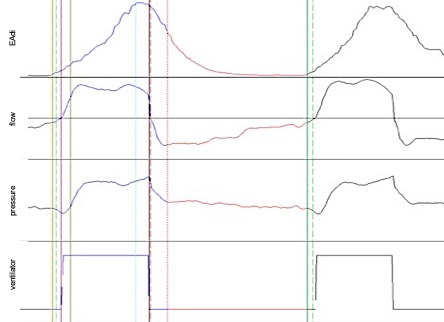


**ESM figure 2:** Tracings and cursors used in the study. For cursor description please refer to “cursor placement”, for definition of times and delays please refer to ESM table 1.

**ESM figure 3**: Questionnaire to assess patient comfort for each ventilation mode; original sheet for first mode, for second mode the sheet is headlined „mode 2“ but otherwise just the same.
Translation: German title of the trial just below the logo of University Medical Center of Goettingen followed by lines to ad patient no. & date of examination. Underlined headline: “patients questionnaire”, just below it “mode 1”
Question 1: Does ventilation via facemask facilitate your breathing? Yes; no
Question 2: Do you feel your exhalation is impaired? Yes; no
Question 3: Does the ventilator react too fast/ too slow? (Does the ventilator adapt to your breathing?) Yes; no
Question 4: Do you get too much/ too little air? Yes; no
Question 5: Do you feel the mask does not seal properly/ too much air leaks? Yes; no


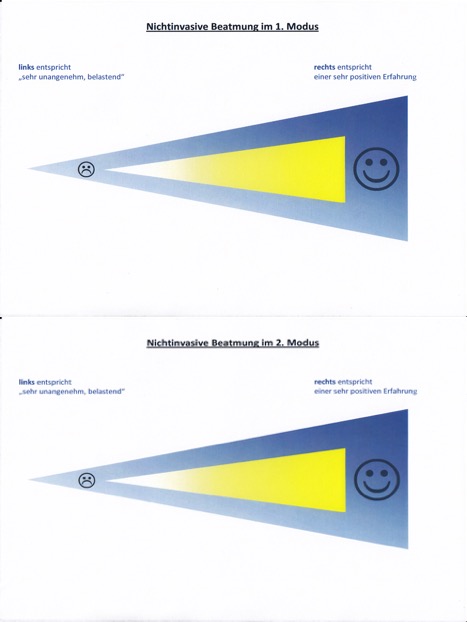


**ESM figure 4**: Visual analog scale to evaluate overall impression of the ventilation mode, each mode was evaluated separately. The patient was given a pen and asked to draw a vertical line at the place where they feel their comfort level is.
Translation: Headline “noninvasive ventilation mode 1/ 2; left means “very uncomfortable, wearing”, right means a very pleasant experience
These explanations were also given to the patient during the trial
